# Circulating Metabolic Markers Related to the Diagnosis of Hepatocellular Carcinoma

**DOI:** 10.1155/2022/7840606

**Published:** 2022-12-09

**Authors:** Da Xie, Guangcong Zhang, Yanan Ma, Dongyu Wu, Shuang Jiang, Songke Zhou, Xuemei Jiang

**Affiliations:** ^1^Department of Gastroenterology, Hainan General Hospital (Hainan Affiliated Hospital of Hainan Medical University), Haikou 570100, China; ^2^Department of Gastroenterology and Hepatology, Zhongshan Hospital of Fudan University, Shanghai 200030, China

## Abstract

Primary liver carcinoma is the sixth most common cancer worldwide, while hepatocellular carcinoma (HCC) is the most dominant cancer type. Chronic hepatitis B and C virus infections and aflatoxin exposure are the main risk factors, while nonalcoholic fatty liver disease caused by obesity, diabetes, and metabolic syndrome are the more common risk factors for HCC. Metabolic disorders caused by these high-risk factors are closely related to the tumor microenvironment of HCC, revealing a possible cause-and-effect relationship between the two. These metabolic disorders involve many complex metabolic pathways, such as carbohydrate, lipid, lipid derivative, amino acid, and amino acid derivative metabolic processes. The resulting metabolites with significant abnormal changes in the concentration level in circulating blood may be used as biomarkers to guide the diagnosis, treatment, or prognosis of HCC. At present, there are high-throughput technologies that can quickly detect small molecular metabolites in many samples. Compared to tissue biopsy, blood samples are easier to obtain, and patients' willingness to participate is higher, which makes it possible to study blood HCC biomarkers. Over the past few years, a substantial body of research has been performed worldwide, and other potential biomarkers have been identified. Unfortunately, due to the limitations of each study, only a few markers have been widely verified and are suitable for clinical use. This review briefly summarizes the potential blood metabolic markers related to the diagnosis of HCC, mainly focusing on amino acids and their derivative metabolism, lipids and their derivative metabolism, and other possible related metabolisms.

## 1. Epidemiology of Hepatocellular Carcinoma (HCC)

Primary liver carcinoma is a global public health challenge. According to the World Health Organization's global cancer statistics, primary liver carcinoma was the sixth most common cancer and the third leading cause of cancer death in the world in 2020, with about 906,000 new cases and 830,000 deaths [1]. In most regions of the world, there are significant gender differences in the incidence and mortality of HCC, with males at least twice as likely as females to suffer from the disease [2]. The incidence of liver cancer ranks fifth globally, while the male mortality rate comes in second [3, 4].

HCC is the most common type of primary liver carcinoma, accounting for 90% of all cases [3]. The incidence of HCC has been declining in high-incidence regions, such as Italy and many Asian countries, and increasing in low-incidence regions, such as India, the Americas, Oceania, and many European countries [5]. The major risk factors for HCC include chronic hepatitis B virus (HBV) and hepatitis C virus (HCV) infections, alcohol intake, and nonalcoholic fatty liver disease (NAFLD) [6–11] related to diabetes or obesity. Among them, alcohol intake has been suggested to be a predictor of cirrhosis in patients with chronic HCV infection and a predictor of death in patients with chronic HCV or HBV infection [12]. Larger tumors and microvascular infiltration have been found in patients with NAFLD-HCC more frequently than in patients with HCV-HCC [13]. Early diagnosis of NAFLD-HCC can lead to curative treatment and improve prognosis [14]. Other less common risk factors for HCC are hepatic cirrhosis caused by primary biliary cholangitis (PBC), hemochromatosis, and *α*1-antitrypsin deficiency [4]. Several measures have been taken over the past decades to reduce the risk of liver cancer, such as hepatitis B vaccination [4, 15], antiviral treatment for hepatitis C [16], and the aflatoxin elimination project (replacing maize with rice as a staple food) [17]. Body mass index, lifelong drinking, smoking, and diabetes-relatedlifestyle-specific metabolic characteristics are thought to be positively correlated with HCC [18]. A meta-analysis has shown that the diagnosis rate of HCC is increasing because of tumor surveillance, particularly semiannual surveillance, and nonviral-multietiological HCC (including metabolic disorders and cryptogenic liver disease) accounts for a larger proportion of HCC diagnoses than other etiologies. With increasing treatment methods for HCC, the priority treatment options of patients with different Barcelona Clinic Liver Cancer (BCLC) stages have changed, and the proportion of patients undergoing resection and radiofrequency ablation has increased. Radiofrequency ablation is a preferred treatment choice, and the use of sorafenib in patients with advanced HCC is increasing. The increases in diagnosis and treatments have improved overall survival in patients with HCC [19]. It is likely that metabolic diseases, such as obesity, diabetes, and metabolic syndrome, which are becoming more prevalent worldwide, will reverse the current progress in controlling liver cancer risk [20]. These results indicate that nonviral HCC should be the focus of diagnosis, treatment, prevention, and control in the future.

## 2. Existing Screening and Diagnostic Methods for HCC and Their Limitations

Based on the molecular classification of gene expression, HCC can be roughly divided into proliferation and nonproliferation categories [21]. HCC intratumor heterogeneity (ITH) and tumor microenvironment (TME) change with the proliferation of tumor cells [22], further increase the difficulty of early diagnosis and treatment of HCC. Currently available noninvasive screening and clinical diagnosis methods for HCC include serum alpha-fetoprotein (AFP) detection combined with abdominal ultrasound (US), enhanced computed tomography (CT), or magnetic resonance imaging (MRI) [4]. Enhanced MRI using gadoxetic acid as a liver-specific contrast medium has been shown to be superior to dynamic enhanced CT scanning or other types of contrast-enhanced MRI scanning in early screening and diagnosis, as well as to US in detecting early tumors [23]. However, the high cost of MRI screening limits its application in HCC monitoring. The new point shear wave elastography (pSWE) and two-dimensional shear wave elastography (2D-SWE) methods provide liver stiffness information for the differential diagnosis of focal liver lesions but still cannot fully replace liver biopsy in the diagnosis of HCC [24]. Unfortunately, serum AFP has low sensitivity for clinical diagnosis and preclinical prediction of HCC [25], while imaging examinations are difficult to use to define the properties of hepatic nodules. The heterogeneity in HCC results in great discrepancy in the targeted treatment and prognosis guided by discontinuous multipoint liver tumor tissue biopsy. Liquid biopsy [26], is an emerging diagnostic tool that can be used to analyze the blood components of cancer patients to obtain potential blood biomarkers. On one hand, liquid biopsy can avoid diagnostic error caused by sampling error of liver biopsy and allows continuously monitoring of TME. On the other hand, liquid biopsy has the advantages of easier specimen acquisition, less trauma, and a high patient willingness to participate. Compared to tissue biopsy, liquid biopsy may have a better prospect of providing a dynamic diagnosis and prognostic basis during tumor genesis and development [27].

At present, AFP is the most studied blood biomarker for the diagnosis of HCC. Compared to lectin-reactive AFP (AFP-L3) and des-gamma-carboxy prothrombin (DCP), AFP has shown the best performance in distinguishing HCC cases from non-HCC controls [28]. The combination of these three biomarkers for early detection of HCC is more common in Asia, while AFP-L3 and DCP have been used for HCC risk stratification in the United States [29]. The diagnostic rate of early HCC has been improved in Japan and Taiwan by combining one or several biomarkers (such as AFP, AFP-L3, and DCP) with liver US, providing patients with early HCC diagnosis and access to radical treatment (surgical resection, liver transplantation, and local ablative therapy), and thus improving their overall survival (OS) [30]. Other serum biomarkers relevant for HCC diagnosis include Dickkopf-p1 (DKK-1), which can be used for the diagnosis of AFP-negative HCC), a-1-fucosidase (AFU, an early HCC marker), nerve growth factor (NGF), which distinguishes between cirrhosis that does or does not develop into hepatocellular carcinoma), oncostatin M (OSM), a cytokine elevated in HCC), and alpha-1 antitrypsin (A1AT), which is elevated in HCC compared with cirrhosis) [31].

Vitronectin (VTN) is a glycoprotein found in the blood and extracellular matrix. The complete amino acid sequence of VTN has been deduced from a human liver cDNA library, and it has high homology in humans, mice, and rabbits [32]. The serum levels of VTN in patients with HCC are significantly higher than those in healthy people [33] or HBV-positive patients with chronic liver disease (cirrhosis and chronic hepatitis) [34, 35]. High levels of VTN have also been detected in the serum of HCV-positive patients with cirrhosis [36, 37]. In addition, serum VTN and AFP have similar differential diagnostic values for HCC and can improve the diagnostic value of AFP alone [33, 34].

MicroRNAs (miRNAs or miRs) are small noncoding RNAs that have been studied for more than 20 years. At present, hundreds of different miRNAs have been detected in blood and tissues, and their expression levels have been found to be dysregulated in a variety of diseases. The dysregulated expression levels of multiple miRNAs related to HCC have shown various clinical correlations [38]. A recent multicenter clinical trial has confirmed that a 12-miR panel (including miR-140, miR-183, miR-30e, miR-103a, miR-12b, miR-93, miR-142, miR-21, miR-29c, miR-424, miR-181a, and miR-340) can be used as a risk assessment tool for detecting gastric cancer and that it shows clinical specificity for seven common cancers, including HCC [39]. However, few clinical studies have assessed HCC-related miRNAs, thus limiting clinical applications. Therefore, the combination of multiple biomarkers appears to have better development prospects than single biomarkers for improving the early diagnosis of HCC.

## 3. Application of Metabolomics in Metabolic Disorders in HCC

**Figure 1:**
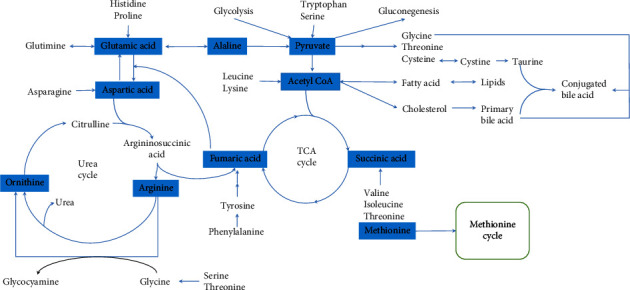
Various metabolic pathways are interrelated and in a state of dynamic equilibrium. TCA cycle, tricarboxylic acid cycle.

Under normal physiological conditions, the body's various metabolic pathways are interrelated and in a state of dynamic equilibrium (Figure 1). When metabolic abnormalities in the body are present, the body can compensate to a certain extent. However, when the compensatory capacity is exhausted, metabolic disorders occur, and the levels of various metabolites become abnormally elevated or decreased. The rapid proliferation of hepatic malignant tumor cells requires the body to provide a large amount of energy in a short period, which will lead to a high level of aerobic glycolysis (“Warburg effect”) [40] and even activate the pentose phosphate pathway (PPP), the hexosamine pathway, mitochondrial oxidative phosphorylation (OXPHOS), and the tricarboxylic acid cycle (TCA cycle). Amino acids, nucleotides, lipids, bile acids, urea, and ammonia may also be involved in this metabolic process [41–48]. These metabolic pathways may be interrelated or interact with each other, leading to varying concentrations of a series of small molecule metabolites in the TME. One or more of these metabolites may be used as a liquid biopsy biomarker for early screening of HCC and distinguishing it from nontumor liver diseases. Metabolomics has been widely used in liquid biopsies and can detect the concentration levels of small metabolites in samples with high throughput in a short period of time. Some potential circulating biomarkers have been found to help diagnose and guide the treatment of HCC, but few candidate biomarkers have been translated into clinical application due to the limited sample size and diagnostic performance.

## 4. Metabolism of HCC in Different Stages

The early and advanced stages of HCC seem to have their own unique metabolic fingerprints. The Kim et al. study has provided a metabolic biomarker portfolio consisting of three amino acids (methionine, proline, and ornithine) and two carnitines (pimelylcarnitine and octanoylcarnitine) using targeted metabolomics analysis based on gas chromatography time-of-flight mass spectrometry (GC-TOFMS) and liquid chromatography-mass spectrometry (LC-MS)/mass spectrometry (MS). This combination is considered to be a potential diagnostic tool for early HCC detection [46]. An *in vitro* experiment has shown that the levels of creatine, acetic acid, and three amino acids (isoleucine, leucine, and phenylalanine) in HepG2 cells were increased compared to normal hepatocytes. This change in amino acid metabolism and enhancement of lipid metabolism can be used to distinguish low-grade metastatic cells from normal cells [44]. Another study has found that serum taurine levels decreased with the progression of tumor node metastasis (TNM) staging in HCC [49]. In a retrospective study using proton nuclear magnetic resonance (1 H-NMR) spectroscopy and multivariate data analysis, untargeted serum metabolomics analysis was performed on blood samples from subjects with early and advanced HCC. The orthogonal partial least squares discriminant analysis (OPLS-DA) showed that serum levels of glucose, lactic acid, lipids, and several amino acids (including alanine, glutamine, 1-methylhistidine, lysine, and valine) were significantly altered. In this study, metabolomics and pathway analyses were combined to identify the metabolic pathways associated with HCC progression, which involved the metabolism of amino acids, pyruvate, and glutamine. It was speculated that the increased metabolism of alanine, glutamate, and lysine was one of the markers of HCC progression and that HCC progression had a significant impact on the metabolism of glycine and serine. The serum tyrosine level is identified as one of the markers of HCC progression. The serum lactic acid level is helpful for the differential diagnosis of early and advanced HCC [45]. A recent 1H-NMR-based metabolomics study has suggested that biomarker combinations including hippurate, creatinine, putrescine, choline, and taurine may be involved in the progression of HCC, while further pathway analysis indicated that taurine and hypotaurine metabolism play a significant role in the occurrence and development of HCC [50].

## 5. Amino Acid Based Metabolic Biomarkers

**Figure 2:**
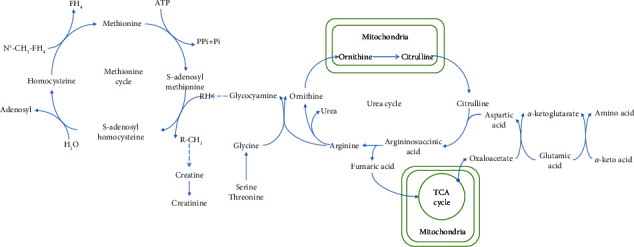
Bridging role of the urea cycle in amino acid metabolism. TCA cycle, tricarboxylic acid cycle; FH4, tetrahydrofolic acid.

The development of HCC is accompanied by amino acid metabolism disorders. A part of the amino acid metabolism pathway is shown in Figures 1 and 2.

The metabolic profiles of patients with HCC 10 years before diagnosis significantly differ from those of normal controls [64]. A significant elevation in serum tyrosine levels has been positively correlated with HCC [65]. Another study not only has reported similar findings but also has observed that isoleucine and glutamine are inversely associated with HCC. This study developed a metabolic risk score based on three amino acids, tyrosine, isoleucine, and glutamine, called the *z* score, which increases as HBV infection progresses to HCC [51]. More significantly, the concentrations of phenylalanine and glutamine can reflect the progression of cirrhosis to HCC, and the liver cancer risk model (R score) constructed by combining age, phenylalanine, and glutamine can be used to classify patients with HCC at 1, 2, or 3 years after baseline [52]. Inhibition of glutamine metabolism improves sorafenib Chemosensitization in oxoglutarate dehydrogenase-like (OGDHL)-deficient hepatoma cells [66]. Decreased glutamine synthesis results in growth inhibition of CTNNB1-mutated HCC xenografts by a combination of glutamine synthetase inhibitors and asparaginase inhibitors [67, 68].

Results from a large multicenter prospective cohort study have shown that glutamine, glutamic acid, *γ*-glutamyl transferase (GGT), and other liver function enzymes were most correlated with AFP, suggesting that the imbalance of specific amino acids and bioamines may be related to the occurrence and development of HCC. The relevance was assessed using Fisher's ratio, with a ratio of less than 3.0 indicating a 4-fold greater risk of HCC than that associated with a normal ratio. Phenylalanine, tyrosine, glutamic acid, glutamine, tryptophan, and kynurenine were positively correlated with the risk of HCC [53].

Other studies have come to similar conclusions. A multicenter study in China has used an LC-MS-based assay to analyze the serum metabolic profiles of 1,448 subjects, including healthy controls and patients with chronic hepatitis B virus infection, cirrhosis, and HCC. A serum metabolic biomarker group consisting of phenylalanyl‐tryptophan (Phe-Trp) and glycocholate acid (GCA) was screened, validated, and defined. This group had a higher diagnostic ability than AFP in differentiating HCC from high-risk patients with cirrhosis, identifying HCC patients with false-negative AFP, screening small HCC (s-HCC) patients from high-risk patients, and distinguishing HCC from other cancers [54]. In summary, the potential of this biomarker group lies in screening patients with preclinical HCC in high-risk populations prior to clinical diagnosis, and its combination with AFP can improve its sensitivity. Another study has also presented a similar opinion. It has used a noninvasive surface-enhanced Raman spectroscopy (SERS) test to detect serum samples of patients with HCC, lung cancers, and breast cancer and found metabolites that could distinguish HCC from the other two cancers, including phenylalanine, tyrosine, proline, tryptophan, and especially phenylalanine [55].

In terms of primary liver cancer classification, studies have found that the differences in serum metabolites between HCC and intrahepatic cholangiocarcinoma (iCCA) are mainly concentrated in seven amino acids, three types of sphingomyelin, and eight types of glycerol, among which the changes in amino acid levels are the most significant. A metabolic biomarker combination of glycine, aspartic acid, sphingomyelin (42 : 3), and sphingomyelin (43 : 2) has been proposed, with an area under the receiver operating characteristic curve (AUC) of 0.890, sensitivity of 75%, and specificity of 90%, which can accurately distinguish HCC from iCCA after verification analysis and biopsy [56]. Dimethylglycine has been found to be significantly elevated in the urine of patients with HCC and to correlate with clinical stage [69].

A recent study has used least absolute shrinkage and selection operator (LASSO) regression analysis to identify an effective method for early detection of HCC progression in cirrhotic patients. This approach utilized a biomarker combination consisting of 11 plasma metabolites and clinical covariates containing AFP. The plasma metabolites included amino acids and their derivatives, such as valine, serine, glycine, isoleucine, and pyroglutamate/glutamate, among which the concentrations of valine, serine, and isoleucine were higher in the plasma samples of patients with HCC [70]. The above studies (Table 1) suggest that amino acids, mainly aliphatic and aromatic amino acids, and especially essential amino acids, might serve as potential HCC biomarkers that need to be confirmed by further studies. Clinical trials are needed to verify their effectiveness. Unfortunately, no clinical trials related to amino acid metabolism and HCC are currently underway.

## 6. Lipid Metabolism Biomarkers

During the progression of chronic liver disease to HCC, the expression levels of various metabolites in plasma change significantly, especially carbohydrates (glucose, galactose, and mannose), amino acids, and lipids (various fatty acids and cholesterol). The metabolic disorder studies have mainly focused on amino acid metabolism and lipid metabolism, in which the amino acid metabolites were up-regulated while the level of linoleic acid was downregulated in HCC patients [57]. This metabolic disorder might be a feature of the progression of chronic hepatitis B (CHB) to HCC, which needs to be confirmed by further research. Other similar studies have also observed that the levels of saturated free fatty acids and saturated lysophosphatidylcholine (LPC) in blood continued to decrease, while the level of saturated triglycerides continued to increase [58]. LPC, acetyl-L-carnitine, and oleic acid amide are expected to become metabolic markers of liver cancer, while LPC has a relatively better diagnostic effect [59]. The reduction in ceramide will be conducive to the growth of HCC. Sphingosine 1-phosphate (S1P) is the most abundant sphingolipid in the liver and plays a role in tumor proliferation, invasion, and angiogenesis. Increasing S1P can support tumor growth [71]. Acylcarnitine is an ester metabolite produced via a biochemical reaction between L-carnitine and fatty acids, which plays an important role in regulating the normal metabolism of carbohydrates and lipids in the human body. It is noteworthy that acylcarnitine and its related metabolites in the blood can be used as potential biomarkers for HCC diagnosis or as targets for drug development [72]. This conclusion provides a valuable reference for the pathogenesis and treatment of liver cancer. In summary, LPC and its derivatives might become potential metabolic biomarkers for HCC. Most of the above studies on LPC used metabolomics methods based on LC-MS and are in the preclinical exploratory stage or clinical detection and verification stage; therefore, a large number of studies will be needed before progression to the clinical trial stage.

## 7. Bile Acid Metabolic Biomarkers

The synthesis and transformation of bile acids involve multiple metabolic pathways (Figure 1). Bile acids are related to obesity, diabetes, NAFLD, and nonalcoholic steatohepatitis (NASH) and are involved in regulating lipid and glucose metabolism through different mechanisms [60, 73, 74]. Dysregulation of bile acid metabolism may be related to HCC and gastrointestinal cancer [75] and may be an early event in the development of HCC [76]. Studies have suggested that bile acids are connected with the risk of virus-associated HCC, in which conjugated primary bile acids with multiplied circulating concentrations are relevant to the increased risk of viral hepatitis-related HCC, while higher levels of secondary bile acids (such as deoxycholic acid) are only related to the reduced risk of HBV-associated HCC [61]. Serum bile acids can affect the tumor immune microenvironment (TIME) associated with the intestinal microbiota, which may have a significant impact on the tumor load and adverse clinical outcomes of HBV-associated HCC patients [77]. As the disease progresses from chronic viral hepatitis B to cirrhosis and then to HCC, the taurodeoxycholic acid level continues to increase while the glycyrrhizin acid level decreases [62]. Therefore, taurodeoxycholic acid and glycyrrhizin acids have the potential to distinguish patients with CHB, LC, and HCC, thereby facilitating the identification of patients with early HCC and access to curative therapy. In one study, differential metabolic analysis was performed on the tissues and serum of HCC patients, and a logistic regression diagnostic model was established. A group of four metabolites consisting of chenodeoxycholic acid (CDCA), LPC (20 : 5), succinyladenosine, and uridine distinguished HCC from cirrhosis with an AUC score of 0.938. The sensitivity and specificity were 93.3% and 86.7%, respectively. Compared to AFP, the diagnostic accuracy of these metabolites was improved to 96.7% and 90.0%, respectively [63]. A clinical trial has proposed that simultaneous elevation of serum bile acid and fibroblast growth factor 19 levels can be used as an indicator of early HCC screening in patients with type-2 diabetes mellitus (T2DM) [60]. The above studies based on mass spectrometry indicate differences in serum bile acid levels among patients with HCC. Although these studies have identified serum bile acid as a candidate biomarker for the diagnosis of HCC, more retrospective studies, prospective studies, or clinical trials are needed to assess its reliability.

## 8. Correlation between Urea Cycle and HCC

The urea cycle plays a vital role in amino acid metabolism (Figures 1 and 2). Pathway enrichment analysis has indicated that defects in the urea cycle, ammonia cycle, and amino acid metabolism may reliably distinguish HCC from cirrhosis. Moreover, the metabolite panel significantly improves the detection of early-stage HCC over that with AFP alone [46]. Significant downregulation of the urea cycle has been observed in the tumor tissues of HCC patients with macrovascular invasion (MaVI). Decreased carbamoyl-phosphate synthetase 1 (CPS1) and gradually decreased carboxylesterase 1 (CES1) levels with increasing vascular invasion may indicate the progression of vascular invasion in HCC [78]. The serum urea level in patients with HCC was significantly higher than that in patients with HBV, HCV, and liver cirrhosis. Multitumor analysis data showed that this was not caused by abnormal renal function, which further provided evidence for distinguishing patients with HCC from those with HBV, HCV, and liver cirrhosis. In addition, the receiver operator characteristic (ROC) curve was used to evaluate the performance of urea, AFP, and carcinoembryonic antigen (CEA) as a single and combined biomarker for HCC, while the combination of the three indicators significantly improved diagnostic efficiency [43]. Based on this, serum urea may be a potential biomarker for HCC; however, few studies have examined the metabolomics of urea in HCC, and no clinical trials are ongoing; therefore, further studies are needed.

## 9. Retinol and HCC

Vitamin A compounds are abundant in the human liver. They include five main metabolites: retinol, retinaldehyde, retinoic acid, retinol palmitate, and *β*-carotene, of which retinol is a bioactive form of vitamin A. Retinol metabolism may also be closely related to the occurrence and development of HCC. One study has observed a negative correlation between retinol and HCC risk [64]. In another study, the metabolomics analysis samples included liver tissue and serum, and the analysis data emphasized that retinol might be a useful biomarker for the diagnosis and prognosis of HCC, while retinaldehyde had a similar biomarker effect on retinol [79]. In this study, a panel composed of retinol and retinaldehyde was considered to be an independent predictor of HCC; the sensitivity and accuracy of HCC detection were higher than those of AFP; and their low expression was correlated with survival time in patients with HCC. Whether retinol can become a potential HCC biomarker remains to be investigated.

## 10. Multibiomarker Model for Monitoring HCC

In 2014, a UK cohort study developed a serum biomarker-based scoring model named GALAD (gender, age, AFP-L3, AFP, and DCP) for the detection and diagnosis of HCC [80]. The GALAD score has high accuracy in diagnosing BCLC stage 0/A HCC [81] and exhibits superior specificity in HCC monitoring in white patients with HBV/HCV infection [82]. Similarly, a case-control study in Germany and a pilot cohort study in Japan have concluded that the GALAD score can detect HCC with high accuracy in patients with NASH with or without cirrhosis [83]. Although the detection ability of ultrasound for HCC is weaker than that of the GALAD score, it can enhance the performance of the GALAD score [84]. Another study has proposed and validated several biomarker combinations containing AFP and protein induced by vitamin K absence-II (PIVKA-II; also known as DCP), but found nonsuperiority to the GALAD score in detecting early and all stages of HCC [85]. A prospective cohort phase III biomarker study has found that GALAD significantly increases sensitivity in HCC monitoring, but the false positive results also increase, thus indicating that GALAD's performance is mediocre and not different from that of the AFP-L3 or Hepatocellular Carcinoma Early Detection Screening (HES) scores alone [86]. The HES algorithm (which combines AFP levels, AFP rate of change, age, alanine aminotransferase levels, and platelet count) has been found to improve the statistical performance of AFP alone in monitoring HCC but underestimate the risk of HCC observed in the next 6 months [87]. Current surveillance strategies lack obvious advantages, but the GALAD score is limited in its general use for all patients with HCC, and its routine application in HCC screening and surveillance may still be unrealistic [88]. A clinical trial (NCT03628651) has proposed and validated that multitarget HCC panels (HOXA1, EMX1, TSPYL5, B3GALT6, AFP, and AFP-L3) are more sensitive at detecting HCC at any stage than the GALAD score and AFP alone [89, 90]. Similar models of HCC monitoring that have outperformed GALAD include the HelioLiver test (methylated free cell DNA markers, AFP, AFP‐L3, DCP, age, and sex), which has also been validated in a clinical trial (NCT05059665) [91]. A new serological scoring tool, the APAC score, which includes parameters such as age, soluble platelet-derived growth factor receptor beta (sPDGFR*β*), AFP, and creatinine, significantly outperforms the GALAD score in identifying HCC in people with cirrhosis, and the diagnostic accuracy of the APAC score is independent of the cause of disease (alcohol, viral infections, and NAFLD); for patients with an HCC grade of BCLC 0/A, the APAC score has high sensitivity and specificity [92]. A multicenter case-control study has indicated that multiple reaction monitoring-mass spectrometry (MRM-MS), including serum biomarker panels for 17 proteins, can distinguish people with HCC from high-risk controls and is significantly more accurate in the early detection of HCC than GALAD scores [93].

## Figures and Tables

**Table 1:** Summary of studies on circulating metabolites associated with HCC.

References	Baseline CLD	Sample type	Metabolite analysis	Metabolites	Correlation with HCC	Metabolite level
[51]	HBV infection	Plasma	1H-NMR	Tyrosine	+	
				Isoleucine	−	
				Glutamine	−	
[52]	Liver cirrhosis	Plasma	1H-NMR and UPLC	Phenylalanine	+	
				Glutamine	−	
[53]	HCC	Serum	UPLC	Panel (tyrosine, glutamate, glutamine, kynurenine, lysine, phenylalanine, and leucine)	+	
	IHBC					
	GBTC					

[54]	HBV infection	Serum	LC-MS	Panel (phenylalanyl‐tryptophan, and glycocholate)	+	Increase
	Liver cirrhosis					
	HCC	ANT, DNT				

[55]	HCC	Serum	SERS	Phenylalanine, tyrosine, proline, and tryptophan (especially phenylalanine)	+	Increase
[56]	HCC	Serum	UHPLC-TOF-MS	Panel [glycine, aspartic, sphingomyelins (42 : 3), and sphingomyelins (43 : 2)]	+	
	iCCA					
	PSC					

[57]	CHB	Plasma	GC/MS	L-serine, cystathionine, creatine, and glycine	+	Increase
	Liver cirrhosis			Linoleic acid		Decrease
	HCC			Panel (L-serine, cystathionine, creatine, and linoleic acid)		

[58]	CHB	Whole blood	UPLC-QTOF-MS	Saturated FFA and saturated LPC		Decrease
	HCC	Liver tissue		Saturated triglycerides		Increase

[59]	HBV infection	Serum	UFLC-IT-TOF/MS	LPC		Decrease
	Liver cirrhosis			Acetyl-L-carnitine		Increase
	HCC			Oleic acid amide		Decrease

[60]	T2DM	Serum	ELISA Standard method	FGF19 TBA	+	Simultaneous increase
	HCC					
	T2DM-HCC					

[61]	HBV-related HCC	Serum	LC-MS/MS	Conjugated primary bile acids deoxycholic acid	+	
	HCV-related HCC				−	

[62]	CHB	Serum	LC-MS/MS	Taurodeoxycholic acid, 1,2-diacyl-3-*β*-d-galactosyl-sn-glycerol glycyrrhizin acid and 5-hydroxy-6E, 8Z, 11Z, 14Z, 17Z-eicosapentaenoic acid		Increase Decrease
	Liver cirrhosis					
	HCC					

[63]	HCC	Liver tissue and serum	UPLC-MS	Panel (CDCA, LPC20:5, succinyladenosine, uridine)	+	
	Liver cirrhosis					

CLD, chronic liver disease; CHB, chronic hepatitis B; GBTC, gallbladder and biliary tract cancers; IHBC, intrahepatic bile duct cancer; ANT, adjacent noncancerous tissue; DNT, distal noncancerous tissue; UPLC, ultra-performance liquid chromatography; UFLC-IT-TOF/MS, ultrafast liquid chromatography coupled with ion trap time-of-flight mass spectrometry; GC/MS, gas chromatography mass spectrometry; GC-SIM-MS, gas chromatography coupled with selected ion monitoring mass spectrometry; UHPLC-TOF-MS, ultra‐high performance liquid chromatography time‐of‐flight‐mass spectrometry; SERS, surface-enhanced Raman spectroscopy; QTOF MS, quadrupole time-of-flight mass spectrometer; LPC, lysophosphatidylcholine; CDCA, chenodeoxycholic acid; FFA, free fatty acids; ELISA, enzyme-linked immunosorbent assay; TBA, total bile acid; and FGF19, fibroblast growth factor 19.

## Abbreviations

HCC: Hepatocellular carcinoma
iCCA: Intrahepatic cholangiocarcinoma
HBV: Hepatitis B virus
HCV: Hepatitis C virus
NAFLD: Nonalcoholic fatty liver disease
BCLC: Barcelona Clinic Liver Cancer
TME: Tumor microenvironment
AFP: Alpha-fetoprotein
US: Ultrasound
MRI: Magnetic resonance imaging
AFP-L3: Lectin-reactive AFP
DCP: Des-gamma-carboxy prothrombin
LC-MS: Liquid chromatography-mass spectrometry
MS: Mass spectrometry
1H-NMR: Proton nuclear magnetic resonance
S1P: Sphingosine 1-phosphate
LPC: Lysophosphatidylcholine
VTN: Vitronectin
GALAD: Gender, age, AFP-L3, AFP, and DCP
HES: Hepatocellular carcinoma early detection screening
sPDGFR*β*: Soluble platelet-derived growth factor receptor beta
APAC: Age, sPDGFR*β*, AFP, and creatinine.

## Additional Points

Many studies worldwide have carried out research on circulating biomarkers for screening and early diagnosis of HCC based on MS and related technologies, and many possible or valuable blood biomarkers have been proposed, mainly in the form of a combination of multiple metabolites. These combinations mainly focus on amino acids and lipids and their derivative metabolic pathways. The metabolism of aliphatic and aromatic amino acids, especially essential amino acids, is closely related to the occurrence and development of HCC. At the same time, lipid metabolism in LPC may be a worthwhile research direction to pursue to investigate metabolic HCC markers. However, due to the differences in baseline characteristics of the enrolled subjects and the limited sample capacity, there are no available metabolic biomarkers that have been widely accepted and incorporated into clinical applications. Other metabolic pathways, such as bile acid metabolism, the urea cycle, nucleotide metabolism, and retinol metabolism, may also be worth pursing in the future. Unfortunately, most of the above metabolites, metabolite combinations, algorithms, or models built based on metabolites are in preclinical exploratory research or clinical detection and verification stages; retrospective or prospective studies are still needed, but very few of them advance to clinical trials. At present, existing liver cancer risk scoring models or algorithms (such as GALAD) cannot be applied in clinical practice because of their inherent limitations. Further research on the reported biomarker algorithms or models or the development of new and different markers is a future development direction in the early screening and diagnosis of HCC. HCC blood biomarkers remain a research challenge in global public health.
